# miR-33a-3p regulates METTL3-mediated AREG stability and alters EMT to inhibit pancreatic cancer invasion and metastasis

**DOI:** 10.1038/s41598-023-39506-7

**Published:** 2023-08-21

**Authors:** Xiaowen Su, Tiantian Lai, Yue Tao, Yong Zhang, Changyong Zhao, Junjing Zhou, Enhong Chen, Maoqun Zhu, Shuo Zhang, Bei Wang, Yong Mao, Hao Hu

**Affiliations:** 1 Department of Hepatobiliary and Pancreatic Surgery, Affiliated Hospital of Jiangnan University, 1000 Hefeng Rd, Binhu District, Wuxi, 214122 Jiangsu Province China; 2 Wuxi Medical College, Jiangnan University, Wuxi, 214122 Jiangsu Province China; 3 Institute of Integrated Chinese and Western Medicine, Affiliated Hospital of Jiangnan University, Wuxi, 214122 Jiangsu Province China; 4 Medical Oncology, Affiliated Hospital of Jiangnan University, Wuxi, 214122 Jiangsu Province China; 5 Hepatobiliary and Pancreatic Surgery, The Third Hospital Affiliated to Nantong University, Wuxi, 214041 China; 6 Medical School, Nantong University, Nantong, 226001 China; 7 Wuxi Institute of Hepatobiliary Surgery, Wuxi, 214122 China

**Keywords:** Cancer, Molecular biology, Oncology

## Abstract

Recent studies have shown that amphoteric regulatory protein (AREG), a member of the epidermal growth factor (EGF) family, is expressed in many cancers and is an independent prognostic indicator for patients with pancreatic cancer, but whether AREG is regulated at the epigenetic level to promote the development of pancreatic cancer (PC) has not been elucidated. Our results support the notion that AREG is overexpressed in pancreatic cancer tissues and cell lines. Functionally, the deletion of AREG impedes pancreatic cancer (PC) cell proliferation, migration, and invasion. In addition, we identified and validated that methyltransferase-like 3 (METTL3) induced the m^6^A modification on AREG and facilitated the stability of AREG mRNA after sequencing. Additionally, we obtained experimental evidence that miR-33a-3p targets and inhibits METTL3 from taking action, as predicted by using the miRDB and RNAinter. Remediation experiments showed that miR-33a-3p inhibits PC progression through METTL3. In summary, this research reveals that miR-33a-3p inhibits m^6^A-induced stabilization of AREG by targeting METTL3, which plays a key role in the aggressive progression of PC. AREG could be a potential target for PC treatment.

## Introduction

Pancreatic cancer (PC) is one of the most aggressive cancers of the digestive system, and its incidence continues to rise worldwide^1^. Despite the improvement of surgical resection, chemotherapy, and radiotherapy, the survival of patients with PC is still poor, with a 5-years survival rate of less than 7%^2^. Postoperative recurrent metastasis is the main determinant of their poor prognosis; however, its mechanism is unknown, and there are few targeted drugs. Therefore, it is important to elucidate the molecular regulatory mechanisms of recurrent metastasis in PC and to develop effective targeted oncologic drugs to improve prognosis.

Amphoteric regulatory protein (AREG) is a multifaceted molecule that functions not only as an extracellular ligand for the EGF receptor (EGFR) but as an intracellular signaling molecule^3^. It is expressed in a variety of tumors, including ovarian, breast, gastric, colon, and PCs. According to studies investigating both hepatocellular and melanoma carcinoma^4,5^, AREG plays a crucial role in cell invasion and metastasis, and AREG promotes ovarian cancer stem cells and drug resistance through the AREG-EGFR-ERK pathway^6^. AREG is involved in regulating the proliferation and migration of erbB2- and HER2-positive breast cancer cells^7^. AREG may serve as a combined marker of prognosis in gastric cancer, and the combined detection of Trop2 and AREG could help predict the prognosis of gastric cancer patients^8^. High AREG mRNA expression is a favorable prognostic biomarker in metastatic colorectal cancer and interacts significantly with bevacizumab^9^. In a prior study, it was revealed that AREG expression was associated with poor prognosis in pancreatic cancer patients^10^. However, the regulatory mechanisms are not fully understood.

Most research has shown that m^6^A is the common and dynamically reversible methylation modification within eukaryotic RNAs, including mRNAs, tRNAs, rRNAs and lncRNAs^11–14^. More than 7000 human transcripts contain at least one m^6^A site within the m^6^A common motif RRACH (R = A/G, H = A/C/U) and the majority of m^6^A sites are enriched in the coding sequence (CDS) and the 3′ non-coding region (3'UTR) of mRNAs, especially near the stop codon^15,16^. Three different types of protein complexes determine the role of m^6^A, including m^6^A "readers" (YTHDC1/2, YTHDF1/2/3, IGF2BP1/2/3), "writers" (METTL3/METTL5/METTL14/METTL16/WTAP/VIRMA/ZC3H13) and “erasers” (FTO/ ALKBH5)^17,18^. METTL3 was the first reported m^6^A writer and was identified as the major methyltransferase for the methylation process. In recent years, studies have proven the many roles and molecular mechanisms of METTL3 in various biological processes, especially in tumors, and in PC, a high level of METTL3 is associated with poor prognosis in patients with PC^19^. In addition, targeting METTL3 has attracted widespread attention as an effective approach to treating various types of tumors^20^.

MicroRNAs (miRNAs) are small non-coding RNA molecules (approximately 22 nucleotides in length) that regulate gene expression by interacting with the 3' untranslated region of genes^21^. miRNA affects various biological processes such as cell growth, metastasis, differentiation, apoptosis, the cell cycle, and tumorigenesis. There is increasing evidence that miRNA dysfunction can interfere with the expression of oncogenic or tumor suppressor target genes, which is related to the pathogenesis of cancer^22^. miRNAs have been widely reported to be aberrantly expressed and to play important roles in the development of cancer, including PC. However, how METTL3 is regulated by miRNAs in PC remains unclear. According to our prior prediction, miR-33a-3p has a targeted regulation of METTL3 expression, and we, therefore, speculate that the miR-33a-3p/METTL3 axis may be an important regulator in the progression of PC.

This study revealed that AREG is expressed at elevated levels in PC tissues and that AREG downregulation repressed PC cell proliferation, migration, and invasion. Bioinformatics predicted a regulatory connection between miR-33a-3p and METTL3-mediated m^6^A modifications. Hence, our research seeks to probe the mechanism of modulation of AREG, METTL3, and miR-33a-3p in PC development.

## Methods

### Ethics approval and consent to participate

This study was approved by the ethical review committee of the Affiliated Hospital of Jiangnan University (LS2021091) and obtained written informed consent from all patients, all experimental protocols used in the study were in accordance with the guidelines of the Declaration of Helsinki.

### Patients and clinical samples

Fresh tumor tissues and paired paracancerous tissue specimens were collected from 36 PC patients who underwent surgical resection from June 2021 to October 2022 at the Affiliated Hospital of Jiangnan University. All patients did not receive preoperative chemotherapy or radiotherapy. Tissue specimens were preserved in RNA Keeper Tissue Stabilizer (Vazyme, China).

### Cell culture

Human pancreatic cancer cell lines (PANC-1, MIA PaCa-2, and BxPC-3) and human pancreatic ductal epithelial cell lines (HPNE) were incubated in high sugar Dulbecco's Modified Eagle Medium (DMEM) medium (HyClone) with 10% fetal bovine serum (Gibco) and grown in a cell culture incubator at 37 °C in 5% CO_2_.

### Stable cell-line construction

A lentivirus encoding a small hairpin RNA (shRNA) targeting METTL3 was created by Gene Chem (Shanghai, China) (targeting sequence: 5′-A TGCACATCCTACTCTTGTAA-3′). Cells (2 × 10^4^) were incubated in 24-well plates. After a 24-h culture, cells were infected with lentivirus at an infection multiple of 10. Stably transfected cells were then selected using puromycin (0.5 μg/mL) (MCE, USA). Analysis of the effect was carried out using qPCR. The qPCR primer sequences are listed in Supplementary Table S1.

### RNA interference and plasmid transfection

Each well was seeded uniformly at a density of (5 × 10^5^) cells in a six-well plate and incubated for 24 h to reach a cell density of approximately 70–80%. For RNA interference, a mixture of 50 nM siRNAs and 8 µL RFect Small Nucleic Acid Transfection Reagent (BAIDAI, China) was incubated for 20 min at room temperature and then added to the cells. For plasmid transfection, a mixture of 10 µg plasmids and 10 µL RFect Plasmid DNA Transfection Reagent (BAIDAI, China) was incubated for 20 min at room temperature and then added to the cells. The mixture was then added to the cultured cells and incubated at 37 °C and 5% CO_2_ for 18–72 h, with a fresh culture change after 4–6 h. RNA and proteins were extracted by transfecting for 48 h. The full length of the METTL3 sequence was inserted into the pcMV3 vector to construct a METTL3 overexpression plasmid (pcMV3-METTL3). Information on the siRNAs used in this study is listed in Supplementary Table S2.

### Quantitative PCR (qPCR)

Total RNA and miRNA were extracted from cells and tissues using the Fast Pure® cells/tissue total RNA isolation kit V2 (Vazyme, China). Total RNA and miRNA were reverse transcribed using the reverse transcription kit (Vazyme, China), according to the manufacturer's instructions. The ABI 7500 Sequence Detection System (Thermo Fisher Scientific, USA) was used with ChamQ SYBR qPCR Master Mix (Vazyme, China) on which real-time PCR analysis was performed. The relative expression of mRNA and miRNA was calculated by the 2-ΔΔCt method and normalized to endogenous GAPDH or U6.

### Cell proliferation assay

The CCK-8 Cell Counting Kit (Vazyme, China) was used to measure cell proliferation according to the manufacturer's instructions. Briefly, cells were spread out on 96-well plates at an initial density of 2 × 10^3^ cells/well. The CCK8 solution was then added to each well at the indicated times and incubated for 2 h at 37 °C. Thereafter, the fluorescence intensity at 450 nm was calculated using a microplate meter.

### Transwell assay

Cells in serum-free medium in suspension (2 × 10^4^–5 × 10^4^ cells/well) were added to the upper chamber (Corning, USA), and medium containing 10% FBS was added to the lower chamber. The plates were incubated at 37 °C and 5% CO_2_ for 24 h. Cells in the upper chamber were taken off with a cotton swab. Cells on the surface of the lower membrane were fixed in a 4% PFA solution for 30 min and stained with 0.1% crystal violet for 15 min. The membranes were then dried and viewed under a microscope. Cells from five random fields of view were counted under a light microscope. For the invasion assay, the test procedure was performed similarly to the migration assay, except that the upper lumen was coated with Matrigel (BD Biosciences, USA).

### Protein isolation and western blotting

The protein was extracted by lysing each group of cells with RIPA (ProteinBio, China). Protein concentration was determined with a Bicinchoninic Acid Assay (BCA) protein analysis kit (Vazame, China). Protein samples were isolated on 10% SDS polyacrylamide gels and then electronically transferred to polyvinylidene fluoride (PVDF) membranes (Millipore, USA). The polyvinylidene fluoride membranes were confined and then incubated with anti-METTL3 (Abcam, ab195352, USA), amphiregulin (Proteintech, 16036-1-AP, USA), E-cadherin (Abcam, ab40772, USA), vimentin (Abcam, ab92547, USA), and GAPDH (ProteinBio, PA11002, China) at 4 °C for one night, followed by incubation with anti-IgG secondary antibodies (abclonal, AS014, USA) at room temperature for 1 h. GAPDH was used as an internal control for protein normalization.

### Luciferase reporter assay

To validate the association of miR-33a-3p with METTL3, the predicted miR-33a-3p binding sequence of the 3 ʹUTR region of METTL3 was wild type (WT), and the corresponding 3 ʹUTR domain of mutant METTL3 was MUT. These segments were inserted into the pmirGlo dual luciferase vector to generate separate WT and MUT METTL3 constructs. The METTL3 of pmirGlo WT or MUT was then co-transfected with miR-33a-3p mimics or miR-negative control (NC) in cells by using the lipofectamine 2000. Forty-eight h later, we measured the double luciferase reporter gene assay kit (Beyotime, China), used the sea kidney fluorophore enzyme as an internal reference, and calculated the ratio.

### RNA immunoprecipitation (RIP)

RIP was undertaken using the Magna RIP RNA-binding protein immunoprecipitation kit (Millipore, MA, USA) following the manufacturer's instructions. In short, cells were collected and lysed with RIP lysis buffer. Magnetic beads were incubated with the METTL3 antibody at room temperature for 1 h. Immunoglobulin IgG was used as a NC. After washing, the spheres were incubated with cell lysis at 4 °C for 3 h. The spheres were gathered and washed, and the RNA composite was isolated by phenol–chloroform extraction. A quantitative polymerase chain reaction was carried out on the RNA-rich segments.

### m6A RNA immunoprecipitation (MeRIP) assay

MeRIP was performed using the Magna MeRIP m6A kit (EPIGENTEK, USA) according to the manufacturer's instructions. Additionally, 2 ug of anti-m6A antibody was simply vortexed with protein A/G beads at room temperature for 90 min at 4 °C. The antibody-coupled pellets were washed three times and then incubated in a mixture of proteinase K and protein digestion buffer. The RNA was next eluted, and the RNA that interacts with it was extracted and measured by qPCR.

AREG Forward 5′-TGAACAGGTAGTTAAGCCCCC-3′

AREG Reverse 5′-GCTGACATTTGCATGTTACTGC-3′

### RNA stability

To measure the RNA stability of PC cells and METTL3 knockdown PC cells, Actinomycin D (MCE, HY-17559, USA) was added to the cells at 5 μg/ml. After incubation for a certain period of time, the cells were collected at different time points, and subsequently, RNA was extracted from each cell sample for real-time quantitative polymerase chain reaction and normalized by the GAPDH method.

### Methylated RNA immunoprecipitation sequencing and RNA sequencing

METTL3 knockout PANC-1 cells and NC cells were constructed as a means to determine m6A modifications of individual genes in PC cells. They were also analyzed using methylated RNA immunoprecipitation (MeRIP) assay-sequencing and RNA sequencing by GeneChem (Shanghai, China).

### Statistical analysis

All data were expressed as mean ± standard deviation (SD). Statistical analysis was carried out using GraphPad Prism 9 software. The *t*-test (two-tailed) and ANOVA were used to examine differences between two groups or between more than two groups. The correlation between AREG and clinicopathological features was analyzed using the Fisher’s exact test. Spearman’s correlation analysis was used to analyze the correlation between METTL3-AREG gene expression. Western blotting and transwell assay results were quantified using ImageJ software. A *P*-value < 0.05 was considered statistically significant (*P < 0.05).

## Results

### AREG is highly expressed in PC and negatively correlates with prognosis

**Figure 1:**
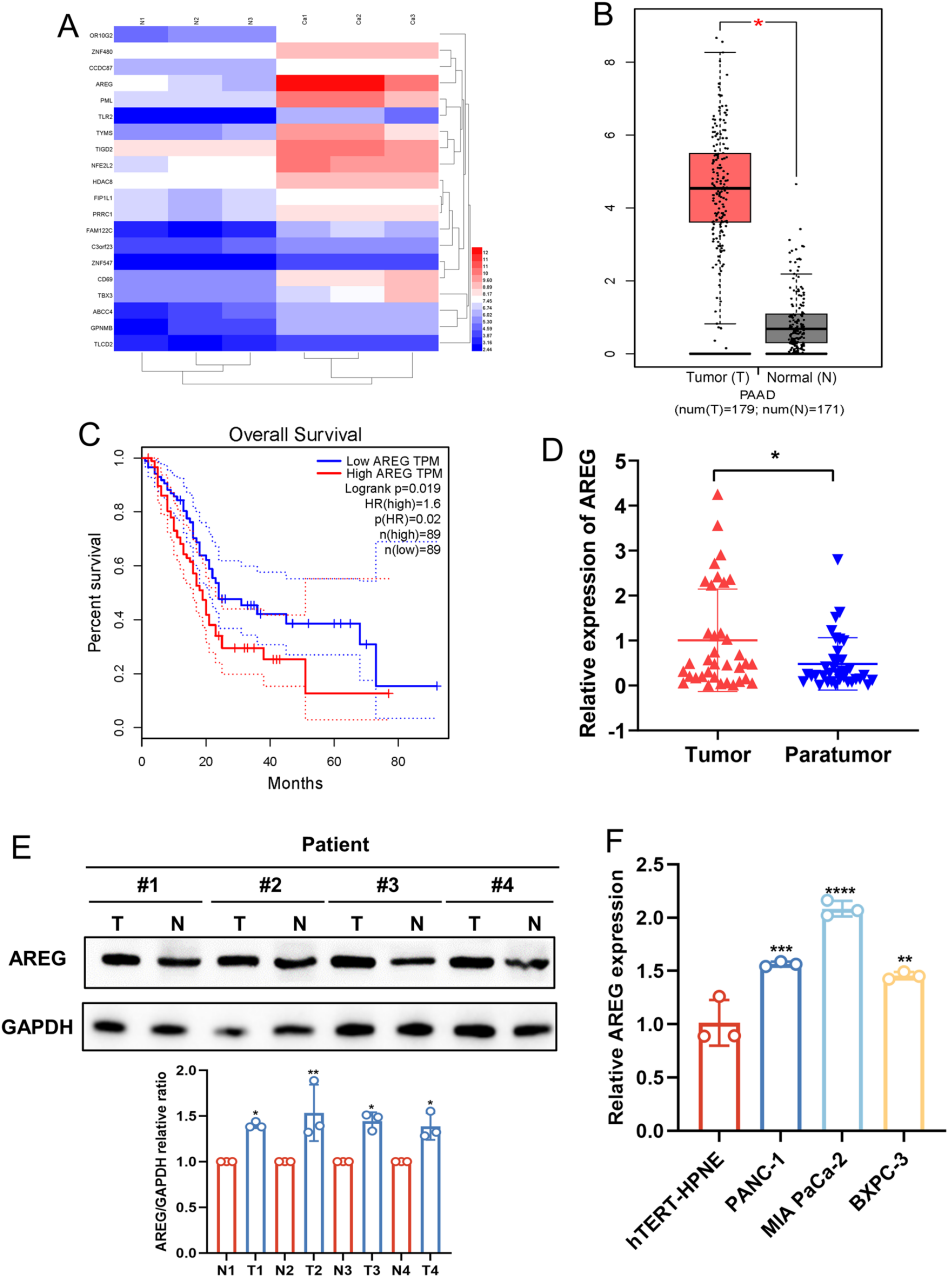
AREG is highly expressed in PC and negatively correlates with prognosis. (**A**) The expression level of AREG mRNA was analyzed using RNA microarray containing 3 pairs of PC tissues and paracancerous tissues. (**B**) AREG mRNA expression was analyzed in PC tissues using an online tool GEPIA. (**C**) The association between AREG mRNA expression and overall survival in PC was analyzed using an online tool Kaplan–Meier plotter. (**D**) AREG mRNA was analyzed by qPCR in 36 pairs of PC tissues collected from our hospital. (**E**) AREG protein expression was examined in four pairs of PC tissues by western blotting. (**F**) AREG expression was examined in PC cells and HPNE cells by qPCR. Data are expressed as the mean ± SD. *P < 0.05, **P < 0.01, ***P ≤ 0.001, and ****P ≤ 0.001 compared with the control. Abbreviations: *PC* pancreatic cancer, *T* tumor, *N* normal, *qPCR* quantitative polymerase chain reaction, *HPNE* pancreatic epithelial cell line.

RNA microarray technology was applied to detect differentially expressed RNAs in three pairs of PC tissues and paraneoplastic non-cancerous tissues. A total of 8345 different genes were identified by strict screening with fold change (FC) ≥ 2 and false discovery rate (FDR) < 0.05. Of these genes, there were 5131 upregulated RNAs and 3214 downregulated RNAs, with AREG expression increasing approximately 30-fold in PC tissues (FC = 39.63, *P* = 0.003, FDR = 0.033) (Fig. 1A). To identify the expression levels of AREG in PC tissues, we first assessed AREG expression in PC tissues using the GEPIA database (http://gepia.cancer-pku.cn/) and observed that AREG mRNA levels were markedly upregulated in PC tissues (T = 179) when compared to adjacent normal tissues (N = 171) (Fig. 1B), and high AREG expression was related to poor prognosis (Fig. 1C). We next analyzed AREG mRNA levels in 36 pairs of PC tissues and revealed that AREG mRNA was upregulated in cancer tissues compared to normal tissues adjacent to the cancer (Fig. 1D). Then, PC tissues were divided into two groups based on the expression level of AREG, and it was found that the level of AREG expression correlated with clinical stage and pathological grade (Table 1). Furthermore, immunoblot analysis confirmed the expression level of AREG protein in PC tissues and paracancerous tissues, which was consistent with expression at the mRNA level (Fig. 1E). In PC cells, we showed that AREG was upregulated at the mRNA level in PC cells compared to pancreatic epithelial cell lines (HPNE) (Fig. 1F). MIA PaCa-2 and PANC-1 (which had a relatively high expression of AREG) were then chosen for the next stage of the research.

**Table 1:** the association of AREG and clinicopathological characteristics^a^.

Characteristics	N of case	AREG level		*P*-value
		Low	High	
Total cases	36	18	18	
Gender (n)				1.000
Male	21	10	11	
Female	15	8	7	
Age (year)				0.711
≤ 60	10	6	4	
> 60	26	12	14	
TNM stage (AJCC)^b^				0.862
I	16	9	7	
II	18	8	10	
III	2	1	1	
T stage				0.045*
T1	10	8	2	
T2	17	8	9	
T3	9	2	7	
Pathological grade (n)				0.018*
G1/G1 ~ G2/G2	27	10	17	
G2 ~ G3/G3	9	8	1	
Lymph node metastasis (n)				0.733
N0	22	10	12	
N1–N2	14	8	6	
Nerve invasion (n)				1.000
No	13	6	7	
Yes	23	12	11	
Vascular invasion (n)				0.691
No	28	13	15	
Yes	8	5	3	

Abbreviations: *N of cases* number of cases, *TNM* Tumor node metastasis, *T stage* Tumor stage.

^a^Fisher’s exact test or Chi-Square test, if appropriate, *P < 0.05.

^b^American Joint Committee on Cancer (AJCC), patients were staged in accordance with the 8th Edition of the AJCC Cancers’ TNM Classification.

### AREG knockdown suppresses proliferation, migration, invasion, and EMT of PC cells in vitro

**Figure 2:**
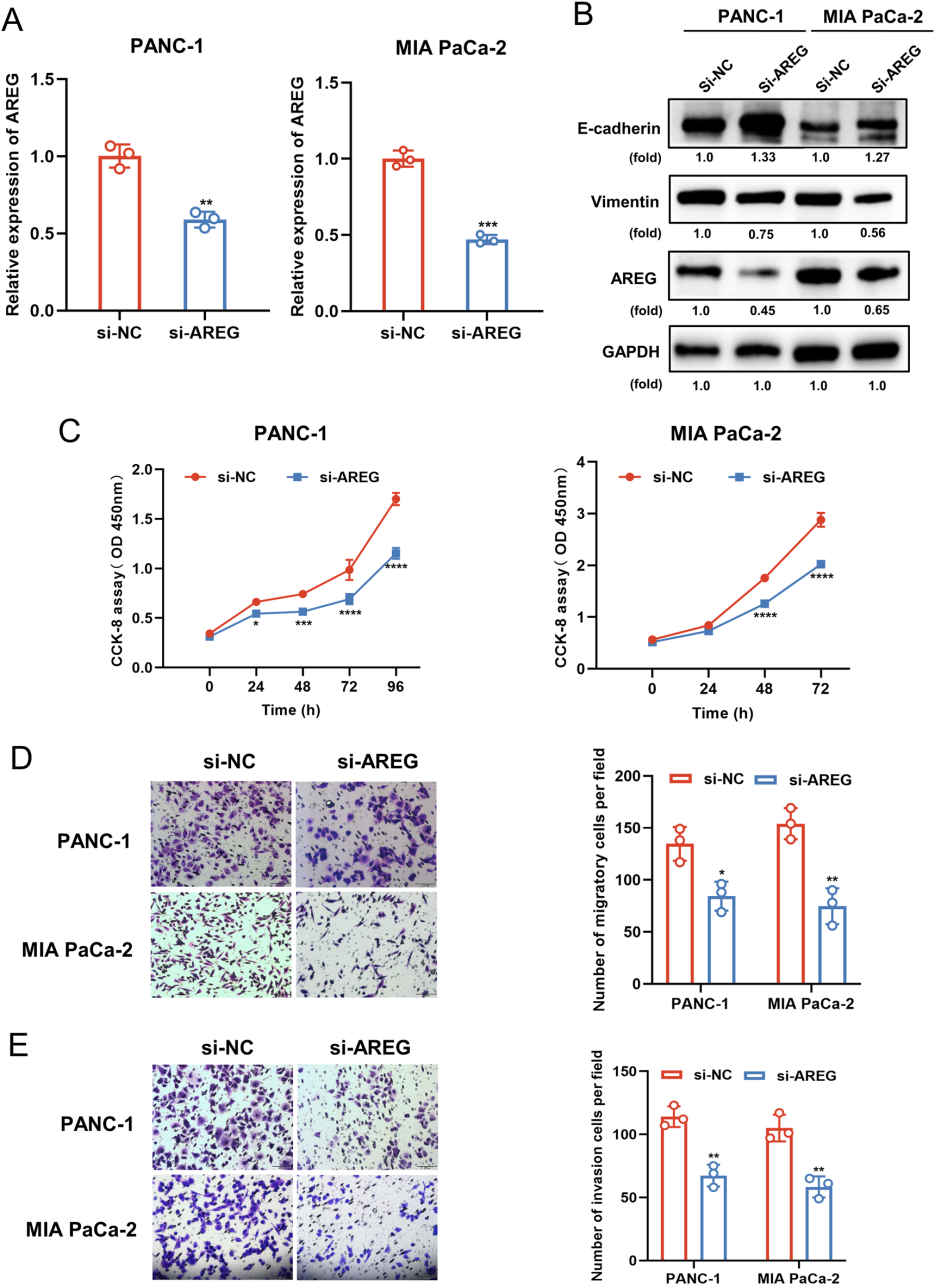
AREG knockdown suppresses proliferation, migration, invasion, and EMT of PC cells in vitro. (**A**) AREG was knocked down using siRNA and the inhibitory efficacy was examined by qPCR. (**B**) The effects of AREG knockdown on the expression of E-cadherin and Vimentin in PC cells were examined by western blotting. (**C**) The effect of AREG knockdown on the proliferative capacity of PC cells was examined by CCK8 assay. (**D**, **E**) The effects of AREG knockdown on the migratory (**D**) and invasive (**E**) capacity of PC cells were examined by transwell assay. Scale bar: 100 μm. Data are expressed as the means ± SD. *P < 0.05, **P < 0.01, ***P ≤ 0.001, and ****P ≤ 0.001 compared with the control. Abbreviations: *PC* pancreatic cancer, *CCK8* cell counting kit-8, *qPCR* quantitative polymerase chain reaction.

Secondly, we explored the biological functions of AREG. qPCR was used to verify that AREG was knocked down in pancreatic cancer cell lines (Fig. 2A) and that the expression of E-cadherin was upregulated and vimentin was decreased in AREG siRNA-transfected cells (Fig. 2B). The silencing of AREG decreased the growth, migration, and invasion of both PC cell lines, as shown by the results of CCK-8 assays and transwell assays (Fig. 2C–E). In general, the downregulation of AREG inhibited PC proliferation, migration, invasion, and EMT in vitro.

### METTL3 regulates m^6^A upregulation on AREG mRNA

**Figure 3:**
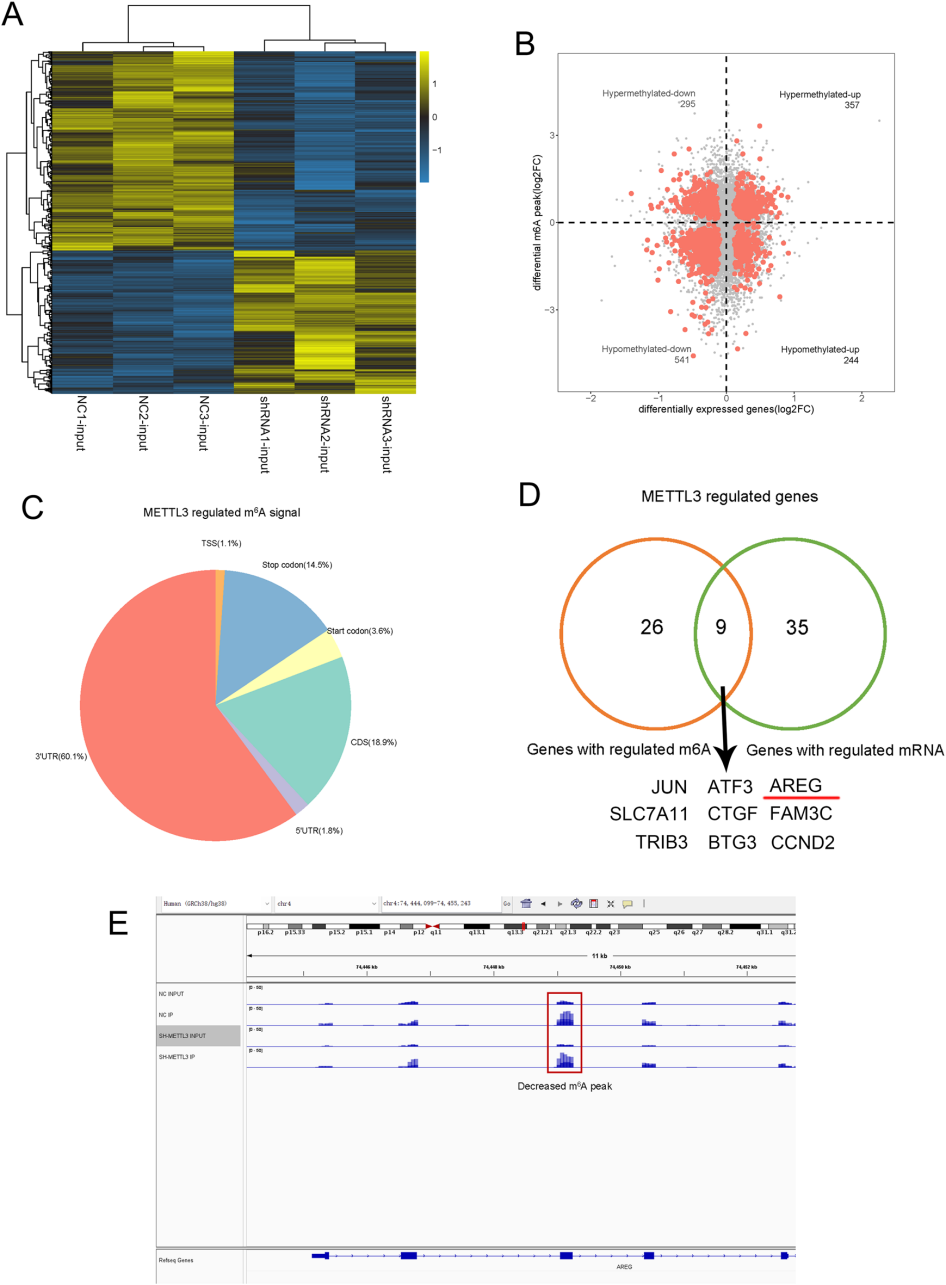
METTL3 mediates m^6^A upregulation on AREG mRNA. (**A**) Differentially expressed genes in PANC-1 cells transfected by NC or METTL3 shRNA were analyzed by RNA-sequencing. (**B**) An interaction analysis of MeRIP-sequencing and RNA-sequencing to show the distribution of m^6^A peaks (fold change > 1) in METTL3 knockdown PANC-1 cells compared to control cells. (**C**) The proportion of m^6^A peaks distributed in the 5′ untranslated regions (5′-UTR), the start codon, the coding region (CDS), the stop codon, and the 3′ untranslated regions(3′-UTR) was calculated. (**D**) Venn diagram was employed toidentify the mRNAs with both changes on m6A peaks and gene expression. (**E**) IGV was used to analyzed the location of m^6^A peak in AREG. Abbreviations: *MeRIP* Methylated RNA immunoprecipitation, *NC* negative control, *m*^*6*^*A* N6-methyladenosine, *5′-UTR* 5′untranslated regions, *3′-UTR* 3′untranslated regions, *CDS* the coding region.

To explore the mechanism of AREG upregulation in PC, we conducted methylation RNA immunoprecipitation (MeRIP) and RNA sequencing on METTL3 knockdown PANC-1 cells and NC cells. Heat map analysis from RNA-seq data listed genes that were differentially expressed in the NC or METTL3 knockdown-treated PANC-1 cells (Fig. 3A). A four-quadrant plot shows that METTL3 knockdown resulted in 357 upregulated hypermethylated RNAs, 295 downregulated hypermethylated RNAs, 244 upregulated demethylated RNAs, and 541 downregulated demethylated RNAs, with a fold change (FC) > 1 (Fig. 3B). And m6A signaling was abundant around the stop codon and 3'UTR of the mRNAs (Fig. 3C). Further screening was carried out to analyze genes with changes in the m6A peak detected by MeRIP-seq (35 genes, fold change > 2) and genes whose expression was upregulated or downregulated in METTL3-deficient PANC-1 cells using RNA-seq (44 genes, fold change > 2), where nine genes, including AREG (Fig. 3D), were overlapping. Then, further analysis showed that the m6A peak in AREG was located in chromosome 4 (74,444,099–74,455,243) (Fig. 3E).

### METTL3-dependent upregulation of AREG mRNA by m^6^A suppressed AREG expression

**Figure 4:**
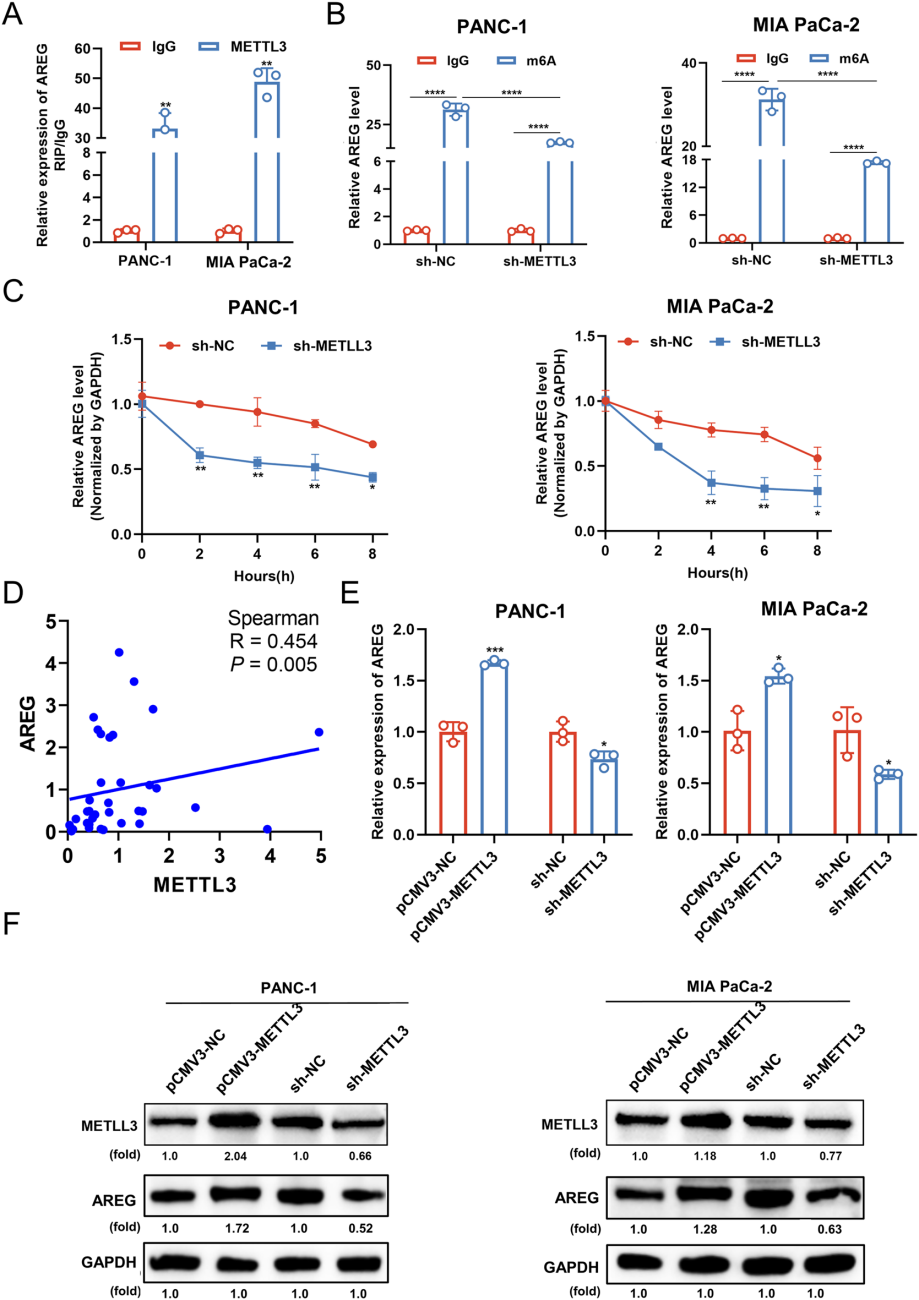
METTL3-dependent upregulation of AREG mRNA by m^6^A suppressed AREG expression. (**A**) RIP assay was used to confirm the binding capacity between AREG mRNA and METTL3 in PC cells. (**B**) The effect of METTL3 knockdown on the m^6^A levels in AREG in PC cells was analyzed by MeRIP-qPCR. (**C**) The effect of METTL3 knockdown on the half-life of AREG in PC cells was examined by qPCR. (**D**) Correlation between AREG mRNA and METTL3 mRNA in PC tissues was analyzed by Spearman. (**E**) The effects of METTL3 overexpression or knockdown on the expression of AREG mRNA were analyzed by qPCR. (**F**) The effects of METTL3 overexpression or knockdown on the expression of AREG protein were analyzed by western blotting. Data are expressed as the means ± SD. *P < 0.05, **P < 0.01, and ****P ≤ 0.001 compared with the control. Abbreviations: *PC* pancreatic cancer, *NC* negative control, *RIP RNA* immunoprecipitation, *MeRIP* Methylated RNA immunoprecipitation, *qPCR* quantitative polymerase chain reaction, *m*^*6*^*A* N6-methyladenosine.

In addition, RIP assay demonstrated that METTL3 bound to AREG mRNA in PANC-1 and MIA PaCa-2 cells (Fig. 4A). MeRIP-qPCR assay indicated that in PANC-1 and MIA PaCa-2 cells, m^6^A levels of AREG mRNA were markedly reduced when METTL3 was knocked down (Fig. 4B). Furthermore, we assessed the rate of RNA decay in METTL3-knocked down PC cells and corresponding control cells. qPCR results showed that the knockdown of METTL3 significantly shortened the half-life of AREG mRNA (Fig. 4C). To confirm the clinical relevance of METTL3 and AREG expression, we performed data analysis on 36 pairs of tissue specimens, and the results showed that AREG mRNA expression was positively correlated with METTL3 mRNA expression in PC (Fig. 4D). Meanwhile, the mRNA and protein expressions of AREG in cells decreased after METTL3 were knocked down, and METTL3 overexpression increased the expression of AREG in cells (Fig. 4E, F). Overall, our data demonstrated that METTL3-mediated upregulation of AREG mRNA m^6^A contributed to the expression and stability of AREG mRNA in PC cells.

### METTL3 was highly expressed in human PC tissues and cell lines

**Figure 5:**
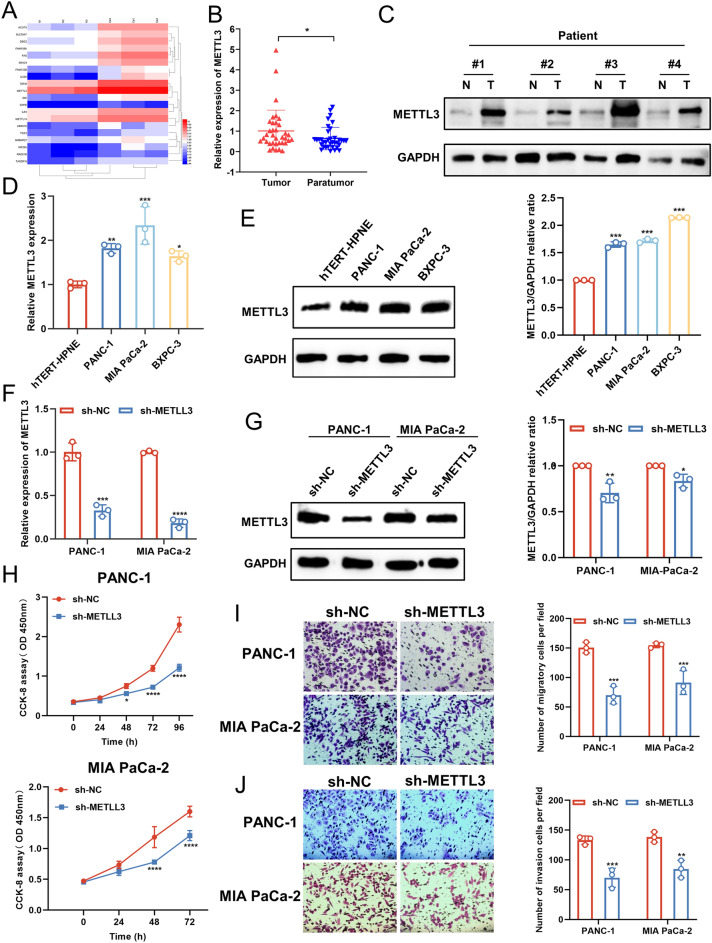
METTL3 was highly expressed in human PC tissues and cell lines. (**A**) The level of METTL3 mRNA was analyzed using RNA microarray in 3 pairs of PC tissues and paracancerous tissues. (**B**) METTL3 mRNA was analyzed in 36 pairs of PC tissues collected from our hospital by qPCR. (**C**) METTL3 protein expression was examined in four pairs of PC tissues by western blotting. (**D**, **E**) The expression of METTL3 in PC cells and HPNE cells was detected by qPCR (**D**) and western blotting (**E**). (**F**, **G**) METTL3 silencing cell lines were established by infecting PC cells with METTL3 shRNA lentivirus. The inhibitory efficiacy was analyzed by qPCR (**F**) and western blotiing (**G**). (**H**) The effect of METTL3 knockdown on the proliferative capacity of PC cells was examined by CCK8 assay. (**I**, **J**) The effects of METTL3 knockdown onthe migratory (**I**) and invasive (**J**) capacity of PC cells were examined by transwell assay. Scale bar: 100 μm. Data are expressed as the means ± SD. *P < 0.05, **P < 0.01, ***P ≤ 0.001, and ****P ≤ 0.001 compared with the control. Abbreviations: *PC* pancreatic cancer, *NC* negative control, *T* tumor, *N* normal, *qPCR* quantitative polymerase chain reaction, *CCK8* cell counting kit-8, *FC* fold change.

The above results indicated that METTL3 medicated the upregulation of AREG mRNA in an m^6^A-dependent manner. We then examined the expression in PC tissues using RNA microarray technology in three pairs of pancreatic cancer tissues and adjacent non-cancerous tissues. As shown in Fig. 5A, several differentially expressed RNAs were identified, among which the expression of METTL3 in PC tissues was increased by approximately threefold (FC = 3.00, *P* = 0.001, FDR = 0.03) (Fig. 5A). Meanwhile, we identified the expression of METTL3 in PC tissues and cells by qPCR and found that METTL3 was expressed at higher levels in 36 pairs of PC tissues than in paired adjacent normal tissues (Fig. 5B). Besides, the level of METTL3 protein was markedly raised in the tissues of representative PC patients compared to normal tissues (Fig. 5C). Consistent with this finding, METTL3 mRNA (Fig. 5D) and protein (Fig. 5E) were expressed at higher levels in three different PC cell lines than in normal pancreatic ductal epithelial cells. These results suggest that METTL3 expression is upregulated in PC. To determine the role of METTL3 in cell proliferation, we depleted METTL3 in PANC-1 and MIA PaCa-2 PC cells by expressing METTL3 short hairpin RNA (ShRNA) (Fig. 5F, G). We showed that depletion of METTL3 reduced the proliferation (Fig. 5H), migration (Fig. 5I) and invasion (Fig. 5J) of these cells.

### METTL3 is a target gene for miR-3a-3p

**Figure 6:**
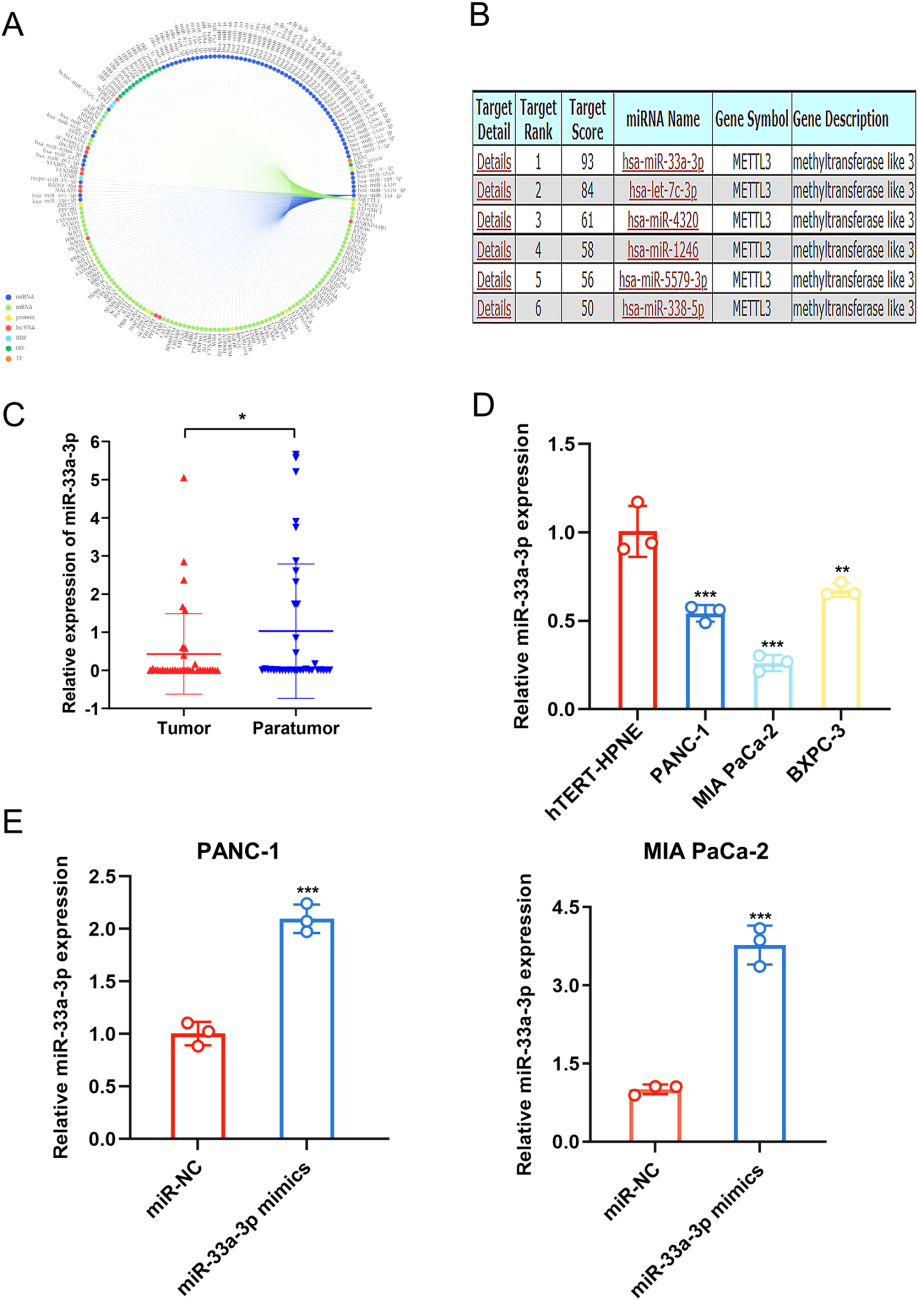
METTL3 is a target gene for miR-3a-3p. (**A**, **B**) The target scores of selected miRNAs that bind to the METTL3 gene. miR-33a-3P was selected from the RNAinter and miRDB databases and predicted to have the highest target scores. (**C**) The expression of miR-33a-3p in 36 pairs of PC tissues collected from our hospital was examined by qPCR. (**D**) The expression of miR-33a-3p in PC cells and HPNE cells was examined by qPCR. (**E**) The overexpression of miR-33a-3p was conducted by transfecting PC cells with miR-33a-3p mimics. And the efficacy was analyzed by qPCR. Data are expressed as the means ± SD. *P < 0.05, **P < 0.01, and ***P ≤ 0.001 compared with the control. Abbreviations: *PC* pancreatic cancer, *NC* negative control, *qPCR* quantitative polymerase chain reaction.

We further explored the upstream mechanisms of METTL3/AREG in PC. As miRNAs are widely recognized as players in cancer progression and regulators of gene expression, potential miRNAs targeting METTL3 were searched through databases such as RNAinter (http://www.rnainter.org/search/) and miRDB (https://mirdb.org/). We found that miR-33a-3p interacted with METTL3 at a high level (Fig. 6A, B). And reviewing the relevant literature, we found few studies on the function and role of miR-33a-3p in PC. Therefore, we selected miR-33a-3p for further study. Given that miR-33a-3p expression levels were significantly lower in PC tissues compared to normal tissues (Fig. 6C). Additionally, we selected PANC-1 and MIA PaCa-2 cells and transfected their mimics for subsequent experiments to explore the potential biological function of miR-33a-3p in vitro (Fig. 6D). As shown in the Fig. 6E, miR-33a-3p expression in PANC-1 and MIA PaCa-2 cells was significantly higher in the miR-33a-3p mock-transfected group than in the miR-NC-transfected group.

### miR-33a-3p downregulates METTL3 expression by directly targeting its 3′-UTR and inhibits proliferation, migration, and invasion

**Figure 7:**
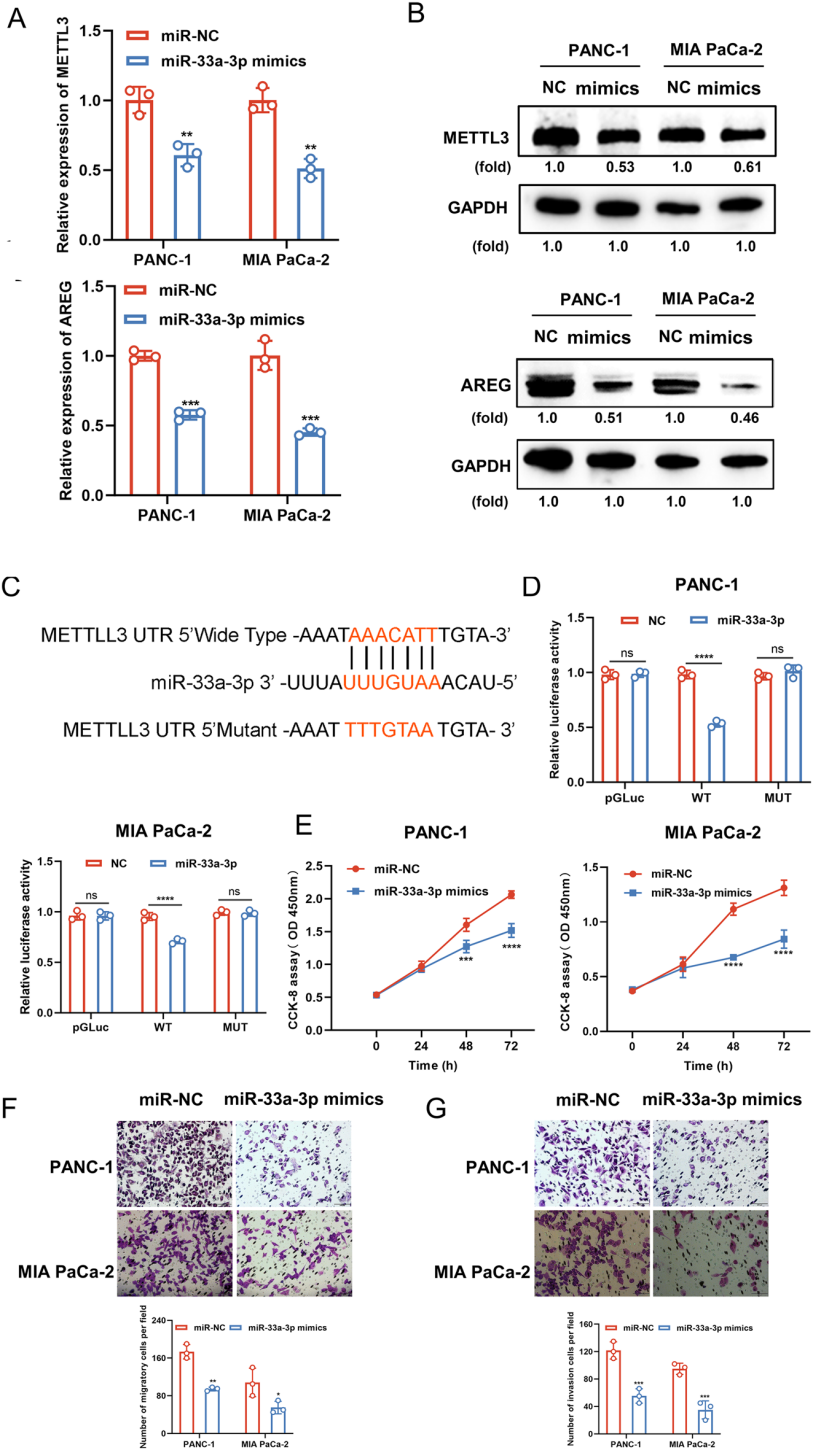
miR-33a-3p downregulates METTL3 expression by directly targeting its 3′-UTR and inhibits proliferation, migration, and invasion. (**A**, **B**) The effects of miR-33a-3p mimics on the expression of METTL3 and AREG were detected in PC cells by qPCR (**A**) and Western blotting (**B**). (**C**) The binding sequence of miR-33a-3p on METTL3 mRNA 3′UTR is indicated. (**D**) Luciferase reporter assay was conducted to examine the binding capacity between miR-33a-3p and METTL3 mRNA 3′UTR in PC cells. (**E**) The effect of miR-33a-3p mimics on the proliferative capacity in PC cells was examined by CCK8 assay. (**F**, **G**) The effects of miR-33a-3p mimics on the migratory (**F**) and invasive (**G**) capacity in PC cells were examined by transwell assay. Scale bar: 100 μm. Data are expressed as the means ± SD. ns *P* > 0.05, *P < 0.05, **P < 0.01, ***P ≤ 0.001, and ****P ≤ 0.001 compared with the control. Abbreviations: *PC* pancreatic cancer, *NC* negative control, *WT* wide type, *Mut* mutatation, *qPCR* quantitative polymerase chain reaction, *CCK8* cell counting kit-8, *5′-UTR* 5′untranslated regions.

We confirmed that transfection of miR-33a-3p mimics in PC cell lines attenuated the mRNA (Fig. 7A) and protein (Fig. 7B) level expression of METTL3 and AREG. To explore the relationship between METTL3 and miR-33a-3p, miR-33a-3p and METTL3 binding sequences and mutant sites are shown (Fig. 7C). Additionally, miR-33a-3p overexpression significantly reduced the luciferase activity of METTL3 WT but not the METTL3 MUT luciferase activity (Fig. 7D). The role of miR-33a-3p in cell proliferation, migration, and invasion in vitro was analyzed using CCK-8 and Transwell assays. The results showed that the miR-33a-3p mock-transfected group significantly inhibited the proliferative capacity of both PANC-1 and MIA PaCa-2 cells compared to miR-NC (Fig. 7E). In addition, miR-33a-3p overexpression significantly reduced the migratory and invasive abilities of cells (Fig. 7F, G). In summary, these data indicate that miR-33a-3p has an inhibitory effect on PC cell growth and metastasis in vitro.

### miR-33a-3p suppresses PC progression via the miR-33a-3p/METTL3 axis

**Figure 8:**
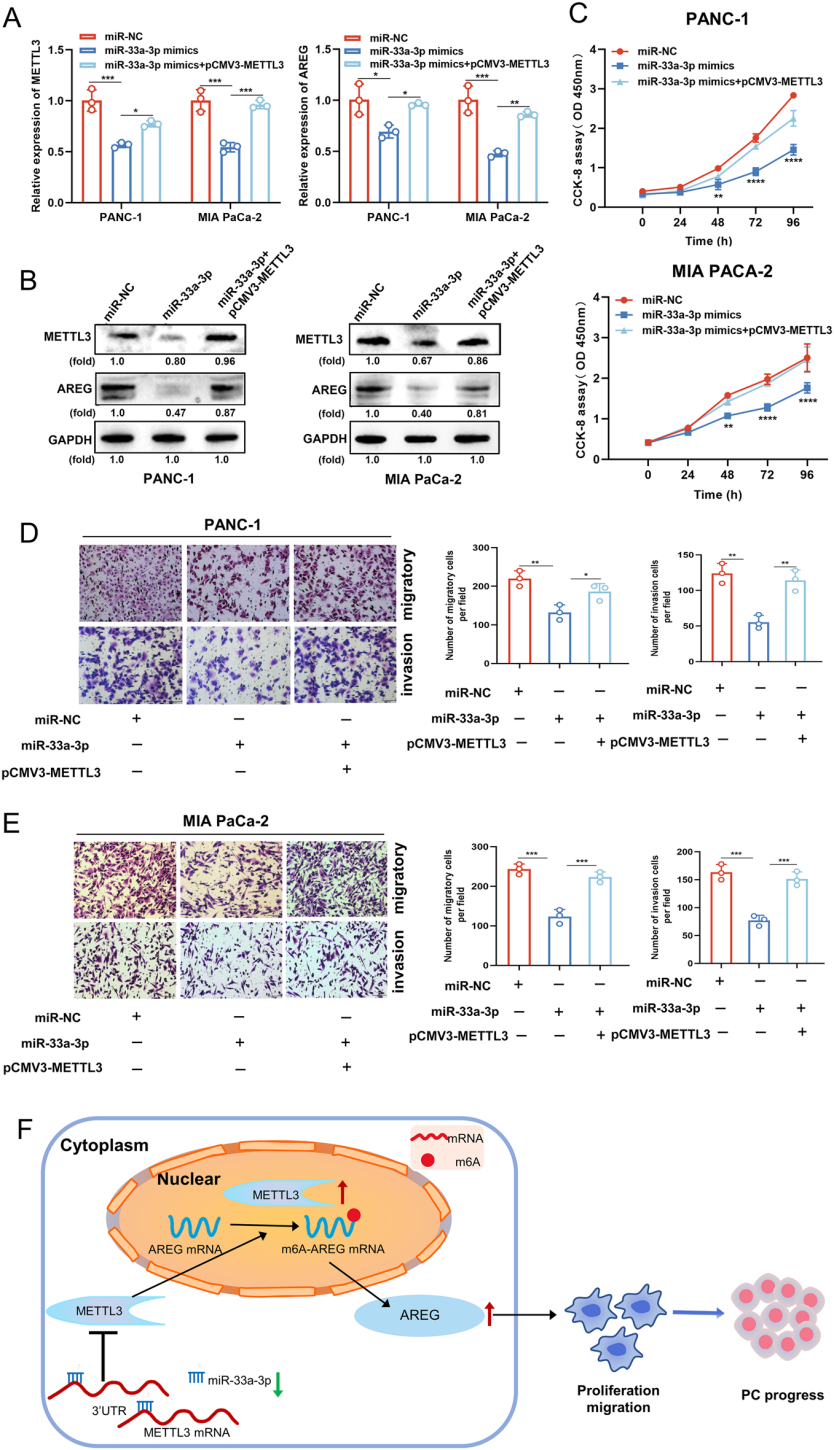
miR-33a-3p suppresses PC progression via the miR-33a-3p/METTL3 axis. (**A**) The reversal effect of METTL3 overexpression on AREG mRNA level in miR-33a-3p mimics PC cells was examined by qPCR. (**B**) The reversal effect of METTL3 overexpression on AREG protein level in miR-33a-3p mimics PC cells was examined by western blotting. (**C**) The reversal effect of METTL3 overexpression on the proliferative capacity in miR-33a-3p mimics PC cells was examined by CCK8 assay. (**D**, **E**) T The reversal effects of METTL3 overexpression on the migratory (**C**) and invasive (**D**) capacity in miR-33a-3p PC cells were examined by transwell assay. Scale bar: 100 μm. (**F**) Schematic model on the role of miR-33a-3p in regulating METTL3-mediated AREG stability in an m^6^A-dependent manner, thus inhibiting pancreatic cancer invasion and metastasis. Data are expressed as the means ± SD. *P < 0.05, **P < 0.01, ***P ≤ 0.001, and ****P ≤ 0.001 compared with the control. Abbreviations: *PC* pancreatic cancer, *NC* negative control, *qPCR* quantitative polymerase chain reaction.

To exploit whether miR-33a-3p exerts its inhibitory effect by regulating METTL3 expression, a rescue assay between miR-33a-3p and METTL3 was performed in PC cells. We found that the expressions of METTL3 and AREG were largely boosted in PANC-1 and MIA PaCa-2 transfected with miR-33a-3p mimics by the introduction of pCMV3-METTL3 at both RNA and protein levels (Fig. 8A, B). CCK-8 assay showed that overexpression of METTL3 significantly reversed of the role of miR-33a-3p mimics on cell proliferation capacity (Fig. 8C). Similarly, transwell analysis showed that overexpression of METTL3 significantly reversed the role of miR-33a-3p mimics on cell migration and invasion capacity (Fig. 8D, E). These results suggest that miR-33a-3p may attenuate the migratory and invasive behavior of PC cells, at least in part, by down-modulating the expression of METTL3.

## Discussion

In short, our findings revealed that low expression of miR-33a-3p in PC was correlated with high expression of METTL3. High expression of METTL3-mediated m6A methylation of AREG enhanced the stability of AREG RNA, led to the increased expression of AREG, and further induced EMT, which promoted the aggressive and metastatic processes of PC (Fig. 8F).

Oncogenic or tumor suppressor genes that are dysregulated in PC have been suggested as potential biomarkers of PC progression. Previous studies have shown that AREG is highly regulated and acts as an oncogene in a range of cancers^23^. AREG has been described as being highly expressed in breast cancer and facilitating tumor progression. In the present study, we explored the role of AREG in PC in the same way and identified that AREG is highly expressed in PC and is strongly related to the prognosis of the disease. Functional assays indicated that silencing of AREG impeded PC cell proliferation, invasion, and migration, but the reasons for its high expression and the molecular mechanism that facilitates tumor progression remain unclear.

Recently, m^6^A modifications in cancer progression have become a focus of research^24^. The most frequent internal mRNA modifications involve N6-methyladenosine (m^6^A), N1-methyladenosine (m^1^A), and 5-methylcytosine (m^5^C)^18^. As the most common catalyst of m^6^A modification, METTL3 has been shown to promote the progression of various cancers, such as breast, lung and gastric cancers^25–28^. It was previously indicated that METTL3 could be induced by m^6^A modification of target mRNAs^29^. Here, we identified m^6^A modifications in AREG by sequencing and hypothesized that AREG overexpression in PC is mediated by its m^6^A modifications. We then confirmed that METTL3 was upregulated in PC cells and positively modulated AREG expression. In addition, we demonstrated that METTL3 promotes m^6^A on AREG mRNA. It is recognized that m^6^A modification can affect mRNA splicing, stability, and translation. Therefore, we identified that METTL3 stabilized AREG mRNA. However, the exact reading protein that recognizes the m^6^A site to better enhance the stability of AREG mRNA needs to be further determined.

The modulation of gene expression in PC by miRNAs has been well documented^30^. Axiomatically, miRNA interacts with mRNA at the 3′UTR to suppress gene expression^31^. In this study, we identified miR-33a-3p as a potential interactor with METTL3 through the miRDB and RNAinter databases. Previously, miR-33a-3p overexpression in hepatocellular carcinoma was found to repress the proliferation and migration of hepatocellular carcinoma cells, indicating that miR-3a-3p functions as a tumor suppressor gene^32^. Therefore, we found that miR-33a-3p could regulate METTL3 by bioinformatics prediction. We further investigated the regulatory relationship between miR-33a-3p and METTL3. Dual fluorescence assays indicate that miR-33a-3p can target METTL3 and inhibit its expression. Finally, the rescue assays showed that miR-33a-3p inhibited PC progression via METTL3. Considering that miRNAs usually have multiple downstream targets, we cannot exclude that miR-33a-3p may also mediate PC progression via other potential targets.

There are some limitations in this study. Firstly, the 36 patients with PC should be followed up for prognosis analysis. Secondly, animal study should be conducted to further confirm the results in vitro.

To conclude, our study reveals that miR-33a-3p inhibits m^6^A-induced stabilization of AREG by targeting METTL3, which plays a key role in the aggressive progression of PC. AREG could be a potential target for PC treatment.

## Conclusion

Our results indicate that miR-33a-3p modulates METTL3-mediated m^6^A-AREG stability and thereby suppresses pancreatic cancer invasion and metastasis. The results of this study help provide new insights for the development of new therapeutic strategies for PC.

### Supplementary Information

Supplementary Information 1.

Supplementary Information 2.

Supplementary Information 3.

## Data Availability

The datasets used and/or analysed during the current study are available from the corresponding author on reasonable request.

## Abbreviations

PC: Pancreatic cancer
AREG: Amphoteric regulatory protein
METTL3: Methyltransferase-like 3
m^6^A: N6-methyladenosine
EMT: Epithelial-mesenchymal transition
CCK8: Cell counting kit-8
MeRIP: Methylated RNA immunoprecipitation
qPCR: Quantitative polymerase chain reaction
DMEM: Dulbecco’s Modified Eagle Medium,
GEPIA: Gene expression profiling interactive analysis
miRDB: MicroRNA target prediction database
RNAinter: RNA interactome database
OS: Overall survival
BCA: Bicinchoninic Acid Assay
3ʹUTR: Three prime untranslated region
siRNA: Small interfering RNA
