# Supplementary material for: miR-33a-3p regulates METTL3-mediated AREG stability and alters EMT to inhibit pancreatic cancer invasion and metastasis
Source: Sci Rep. 2023 Aug 21;13:13587. doi: 10.1038/s41598-023-39506-7 (PMC10442451; doi:10.1038/s41598-023-39506-7)
Supplement: Supplementary file 3 — Supplementary Information 3. [file 41598_2023_39506_MOESM3_ESM.docx]

Table S1. The primer sequences for qPCR.

| Primer | | Sequence 5’-3’ |
| --- | --- | --- |
| GAPDH | Forward | GAAGGTGAAGGTCGGAGTC |
|  | Reverse | GAAGATGGTGATGGGATTTC |
| METTL3 | Forward | GCTTGGTTGGTGTCAAAGGAAAT |
|  | Reverse | TCTGGTTTATGACTGGTGGAACG |
| AREG | Forward | TGAGATGTCTTCAGGGAGTG |
|  | Reverse | AGCCAGGTATTTGTGGTTCG |
| miR-33a-3p | Forward | GCAATGTTTCCACAGTGCATCAC |
|  | Reverse | 试剂盒内包含 |
| U6 | Forward | CTCGCTTCGGCAGCACA |
|  | Reverse | AACGCTTCACGAATTTGCGT |

Table S2. mimics and siRNA sequences used in this study.

|  | | Sequence 5’-3’ |
| --- | --- | --- |
| miR-33a-3p  mimics | sense | CAAUGUUUCCACAGUGCAUCAC |
|  | antisense | GUGAUGCACUGUGGAAACAUUG |
| AREG si-1 | sense | GGAUUUGAGGUUACCUCAATT |
|  | antisense | UUGAGGUAACCUCAAAUCCTT |
| AREG si-2 | sense | GAACGAAAGAAACUUCGACAATT |
|  | antisense | UUGUCGAAGUUUCUUUCGUUCTT |
| AREG si-3 | sense | CACUGCCAAGUCAUAGCCAUATT |
|  | antisense | UAUGGCUAUGACUUGGCAGUGTT |
